# Program Evaluation of the Family Adoption Program for Medical Graduates in India: A Strengths, Weaknesses, Opportunities and Challenges (SWOC) and Stakeholder Analysis in a Medical College in Assam

**DOI:** 10.7759/cureus.100365

**Published:** 2025-12-29

**Authors:** Sthapana Sharma, Sonali G Choudhari, Deepjyoti Das, Ashfia Habib, Jutika Ojah

**Affiliations:** 1 Department of Community Medicine, Gauhati Medical College and Hospital, Guwahati, IND; 2 Department of Community Medicine, Jawaharlal Nehru Medical College, School of Epidemiology and Public Health, Datta Meghe Institute of Higher Education & Research, Wardha, IND

**Keywords:** community medicine, family adoption program, medical education, mixed-methods study, program evaluation, stakeholder analysis

## Abstract

Introduction: The Family Adoption Program (FAP), launched by India's National Medical Commission in 2019, connects medical undergraduates with families to understand community health challenges and social determinants affecting health in Assam. The aim of the study was to evaluate FAP through Strengths, Weaknesses, Opportunities and Challenges (SWOC) and stakeholder analysis at a medical college in Assam.

Methods: A mixed-method evaluation was conducted among 150 MBBS students, 150 adopted families, faculty, health workers, and community leaders from June to October 2025 using validated Google Forms, interviews, and focus group discussions.

Results: Among 150 students, 148 (98.6%) reported positive engagement and 146 (97.2%) cited the FAP as their first field-based exposure. All faculty (100%) supported FAP's Competency-Based Medical Education (CBME) alignment, though four (33.3%) students noted increased workload. Healthcare workers appreciated the gains in community awareness but highlighted communication gaps and supply shortages. Families valued health education but desired continuity and access to screening tools. The SWOC analysis identified strong engagement and community acceptance as strengths, while training gaps and logistical constraints were the primary challenges.

Conclusion: FAP effectively develops compassionate, community-aware physicians. Structured training, essential medical supplies, clear communication protocols, and stakeholder involvement can strengthen FAP as a model for community-based medical education across India.

## Introduction

Community as a classroom is an experiential concept in medical education, as introduced by the National Medical Commission (NMC) [1] to provide undergraduates insight into how living conditions influence public health. The NMC implemented curricular changes through the Family Adoption Program (FAP), launched in August 2019 [2], for all Indian Medical Graduates from the 2021-22 batch under the Competency-Based Medical Education (CBME) curriculum. This initiative addresses the gap in real-world physician experience and aims to improve healthcare delivery in rural India. Community-based medical education offers multiple benefits: developing patient-oriented perspectives, enhancing knowledge acquisition, improving psychomotor skills and communication abilities, and providing exposure to rural general and family medicine practice [3]. The FAP equips medical graduates to function effectively as leaders, clinicians, lifelong learners, facilitators, and researchers while exposing them to public health realities, local health beliefs, and alternative medicine practices alongside allopathy [4]. This study evaluates the FAP using Strengths, Weaknesses, Opportunities and Challenges (SWOC) analysis to examine internal dynamics (strengths, weaknesses) and external factors (opportunities and challenges), contributing to curriculum improvement and program success. 

Aims and objectives

The present study aims to evaluate the FAP for medical graduates in India by conducting a detailed assessment with regard to strengths, weaknesses, opportunities, and threats along with a stakeholder analysis in a medical college in Assam. The study seeks to understand how the program is functioning, what its strengths and weaknesses are, and what opportunities and challenges exist in its current implementation. As part of this evaluation, the study explored the perspectives of different stakeholders involved in or affected by the program. This includes examining their roles, interests, level of influence, and the power dynamics that shape the functioning and outcomes of the FAP. Through this narrative exploration of stakeholder experiences and program processes, the study intended to identify what is working well and where improvements are needed. Ultimately, the study aims to generate practical and actionable recommendations that can strengthen program outcomes, ensure better utilization of available resources, and enhance the engagement and satisfaction of all stakeholders associated with the FAP.

## Materials and methods

The present study is designed as a program evaluation that employs a mixed-methods approach, integrating both qualitative and quantitative techniques to comprehensively assess the FAP. The evaluation was conducted among various stakeholders associated with the program in the Department of Community Medicine, Gauhati Medical College and Hospital (GMCH). These stakeholders include MBBS students across different phases of training, adoptive families, faculty members, medico-social workers, health workers, and representatives of the local village administration.

The study was carried out over a period of five months, from June to October 2025. Participants included were MBBS students from Phase 1, Phase 2, and Phase 3 (Part 1), along with faculty and staff of the Department of Community Medicine, health workers posted in the study area, members of the adopted families, and community leaders from the villages where the program is operational. Only those individuals who provided informed consent were included in the study, while those unwilling to participate were excluded.

A structured sampling strategy has been adopted to ensure adequate and representative coverage of students and families. Within the program, 200 students are allotted to each phase of the MBBS course, giving a total population of 600 students. A two-stage simple random sampling method was used. In the first stage, 50 students were selected randomly from each of the three phases, yielding a final sample of 150 students. In the second stage, each selected student was randomly assigned one family from the pool of five families allotted to their respective phase. As a result, 50 families were adopted by students from each phase, giving a total of 150 families selected for evaluation. This approach ensures balanced representation across the three phases of medical education and strengthens the validity of the assessment by maintaining randomization at both the student and family levels, thereby minimizing selection bias in the SWOC analysis.

Other stakeholders in the program, such as all faculty members and medical social workers from the Department of Community Medicine, health workers involved in the FAP activities, and local community leaders from the study area, were also included. Thus, the final sample comprises 150 students, 150 families, all departmental faculty and staff associated with the program, members of the village administration, and health workers from the selected communities.

Data collection was carried out using a combination of structured schedules, key informant interviews, and focus group discussions. A pre-designed and pre-tested Google Form was administered to the 150 selected students to capture their perceptions of the program. Similar pretested Google Forms will be distributed to all faculty members and medico-social workers engaged in the FAP. Focus group discussions, guided by a standardized FGD guide, were conducted with health workers and local community leaders in groups of eight to ten participants. In-depth interviews were carried out among members of the adopted families using a validated interview schedule to gain deeper insights into their experiences and expectations. This mixed-method approach helped generate a comprehensive and nuanced understanding of the program’s performance and the perspectives of all involved stakeholders.

## Results

**Figure 1:**
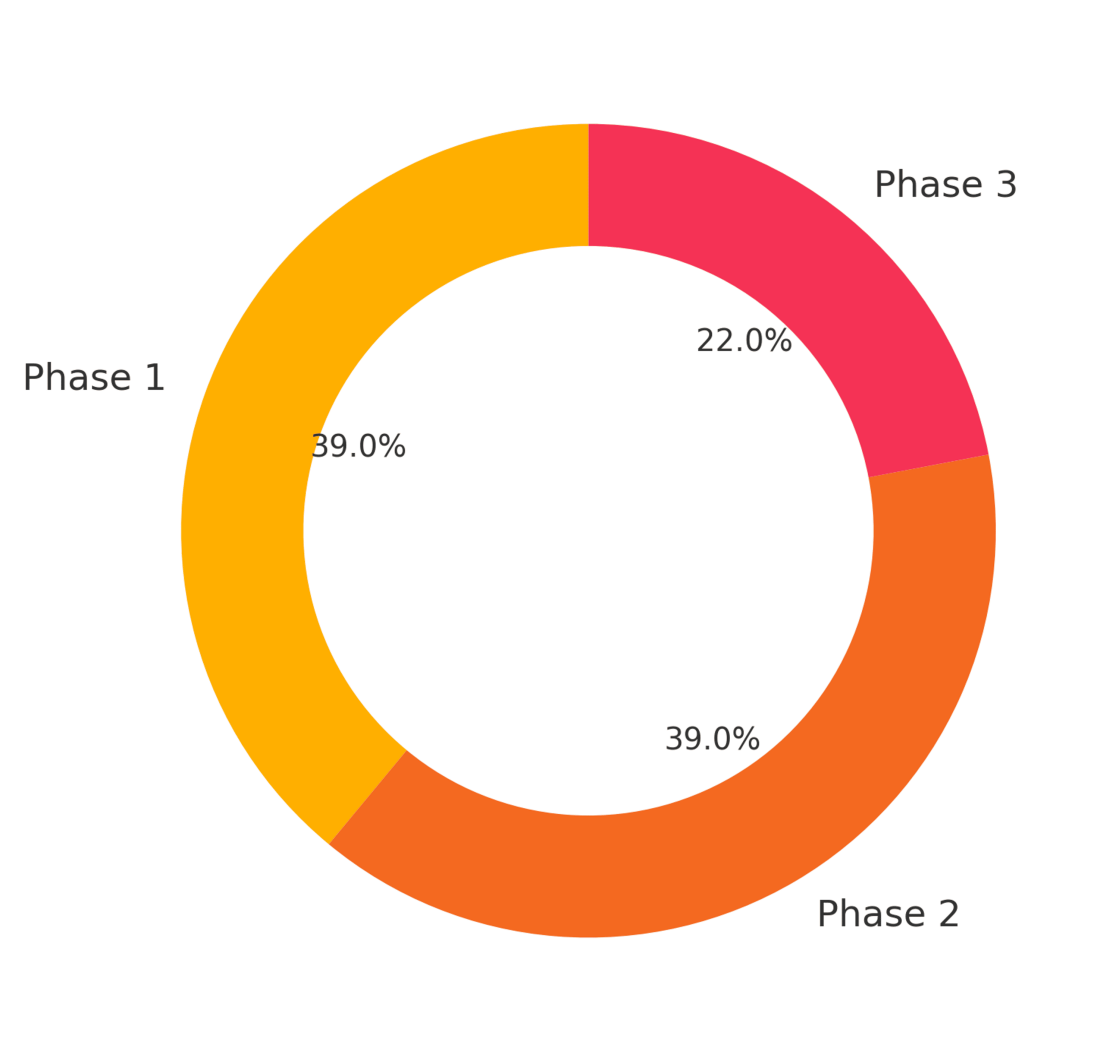
Donut diagram showing student participation with respect to Phases 1, 2 and 3

**Figure 2:**
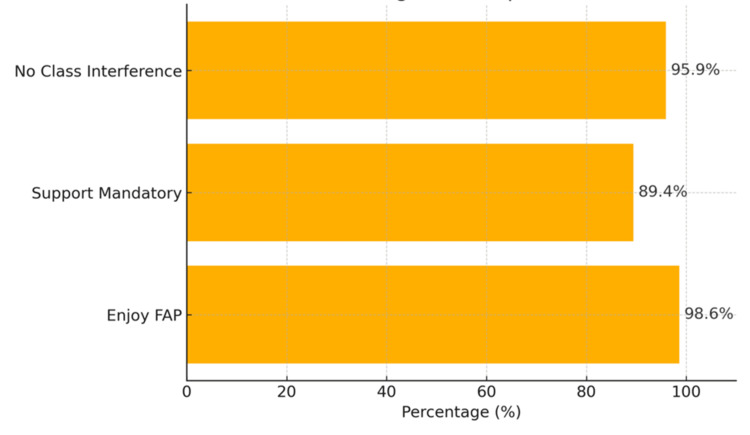
Horizontal bar chart showing the program reception among the students FAP: Family Adoption Program

**Figure 3:**
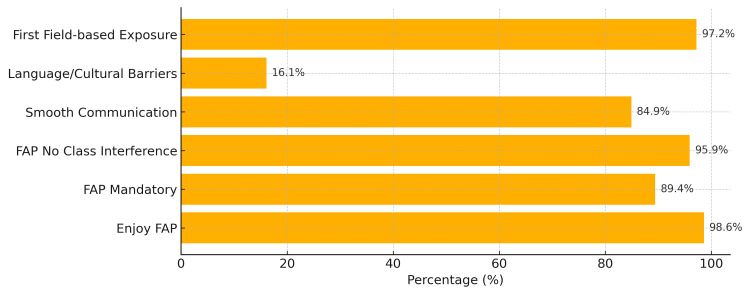
Horizontal bar chart showing students’ perspective of the Family Adoption Program

A total of 150 students participated in the study, representing all three phases of the MBBS program. Among them, 58 (39.0%) were from Phase 1, 58 (39.0%) from Phase 2, and 34 (22.0%) from Phase 3. Most of the participating students reported completing between five and 10 visits to their adopted families, with the majority adopting two to three families, as illustrated in Figure 1. Overall, the program was well-received by the students. Nearly all participants, 148 (98.6%), expressed enjoyment of being part of the FAP, and 134 (89.4%) felt that the program should be made mandatory for all students. Importantly, 144 (95.9%) reported that the field visits and activities under the program did not interfere with their regular academic schedule, as shown in Figure 2. Students also reported significant learning benefits from their participation in the FAP. Most rated their development of key skills, such as communication, understanding social determinants of health, empathy, and teamwork “good” to “very good.” These responses reflect a positive educational impact aligned with the objectives of community-based medical training. While most students, 127 (84.9%), reported smooth communication with their adopted families, a smaller proportion, 24 (16.1%), encountered barriers related to language differences or cultural variations. Despite these challenges, the overall experience remained constructive. For many students, the program served as their first direct exposure to field-based public health practice, with 146 (97.2%) confirming that the FAP was their initial hands-on interaction with community settings as mentioned in Figure 3. Most participants felt that this experience meaningfully strengthened their connection with society and enhanced their understanding of their role as future healthcare professionals.

**Figure 4:**
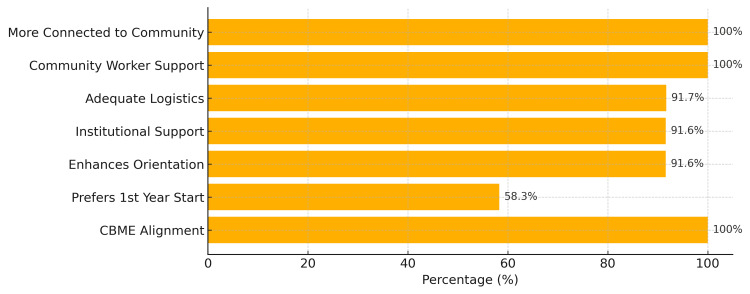
Horizontal bar chart showing faculty perspective on the Family Adoption Program

The FAP survey analysis reveals strong institutional support with 12 (100%) showing faculty involvement and unanimous CBME alignment. Among the 12 respondents, seven (58.3%) have over 10 years of experience, with nine (75%) serving as Group Leaders. Faculty overwhelmingly prefer initiating the FAP in the first year (7; 58.3%), followed by the second year (4; 33.3%). Regarding program impact, 11 (91.6%) agree or strongly agree that the FAP enhances community orientation; four (33.3%) respondents agree that it increases workload as mentioned in Figure 4. Seventy-five percent (9 out of 12) of students agreed that they needed better training before the visits. Institutional support receives positive ratings with 11 (91.6%) showing agreement, and logistic support is adequate for 11 (91.7%) of respondents. Community workers are universally cooperative 12(100%), and 12(100%) of faculty feel more connected to the community post-implementation. For program continuation, 8 (66.7%) recommend proceeding as-is, while 4(33.3%) suggest modifications. Most faculty, 7 (58.3%), report that the FAP doesn't hamper routine activities. Overall, the data indicate strong faculty endorsement of the FAP with recognition of its community engagement benefits, though workload concerns and training improvements warrant attention.

The focused group discussions held with healthcare workers, including Accredited Social Health Activists (ASHAs) and Auxiliary Nurse Midwives (ANMs), provided valuable insights into the functioning and community perception of the FAP. Three major themes emerged from the analysis.

**Figure 5:**
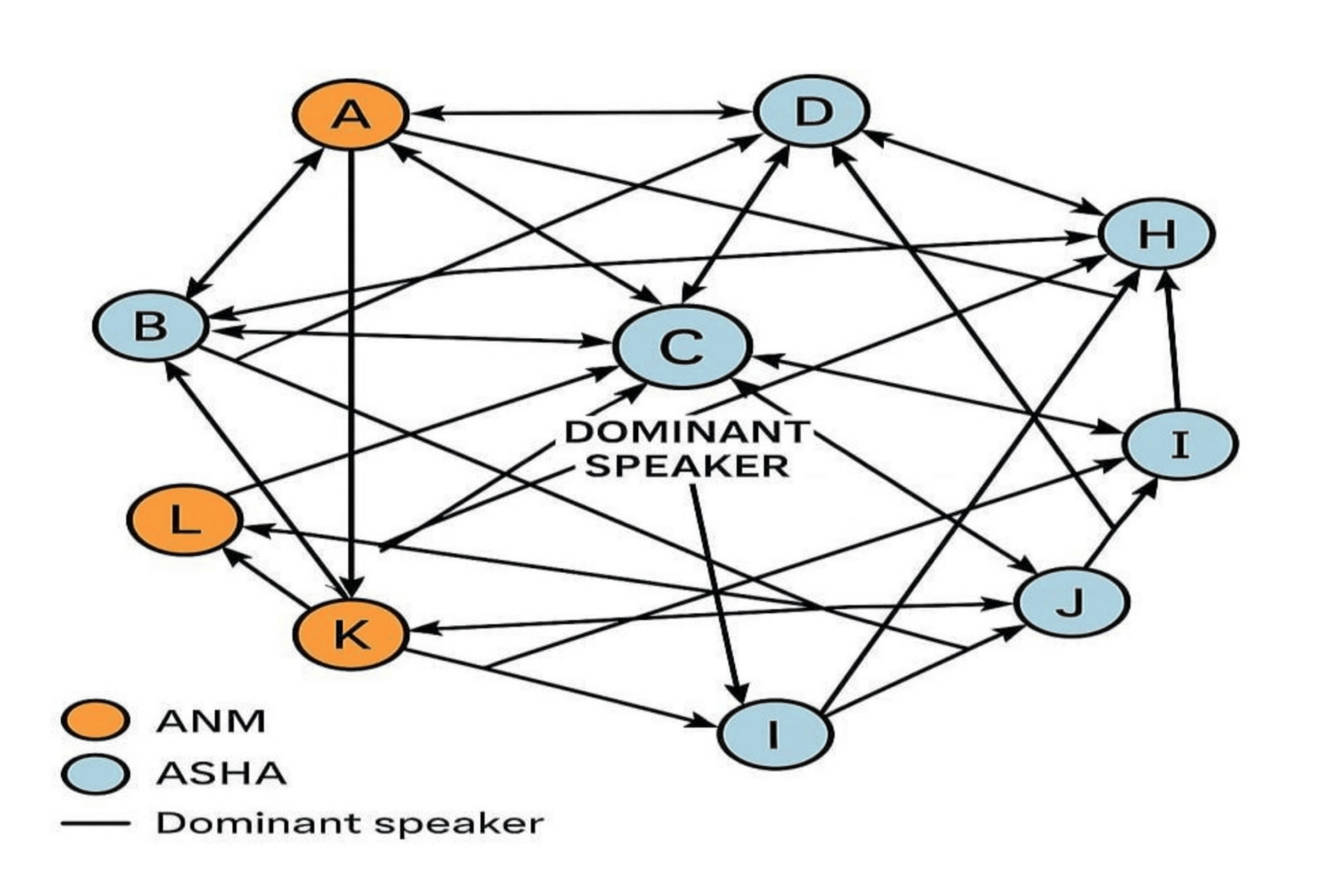
Sociogram of FGD analysis from the healthcare workers (ASHA and ANM). Letters signify participants The orange circles represent the ANM (Auxiliary Nurse Midwife) and the other colored circles represent ASHAs (Accredited Social Health Activists), these were the participants for the Focus Group Discussion (FGD).

Healthcare workers consistently highlighted the positive impact of the FAP on community awareness. They noted that the regular visits by medical students had strengthened understanding of preventive health practices among families, and the presence of students was generally well-received by the community. Many participants felt that the program helped build rapport between the health system and households. At the same time, several gaps and unmet expectations were also identified. One of the key concerns raised was the lack of essential support materials during field visits. Healthcare workers felt that students could improve community trust by carrying and distributing basic items such as paracetamol and oral rehydration salts. Communication barriers were another recurring issue, with many participants reporting difficulty in coordinating with students because of their academic schedules and limited availability. Additionally, healthcare workers expressed that students often did not provide adequate support during hospital visits and were not consistently able to assist families at the point of care. A third important theme related to the role of community leadership and institutional linkage. Participants emphasized that the absence of support from local leaders, including the Panchayat, reduced community ownership of the program. This lack of involvement, they felt, affected the long-term sustainability and perceived authority of FAP activities within the villages. Overall, the discussions highlighted that while the FAP has generated meaningful community engagement and awareness, strengthening support mechanisms, coordination, and local leadership involvement would significantly enhance its effectiveness. All of the above findings are mentioned in Figure 5.

**Figure 6:**
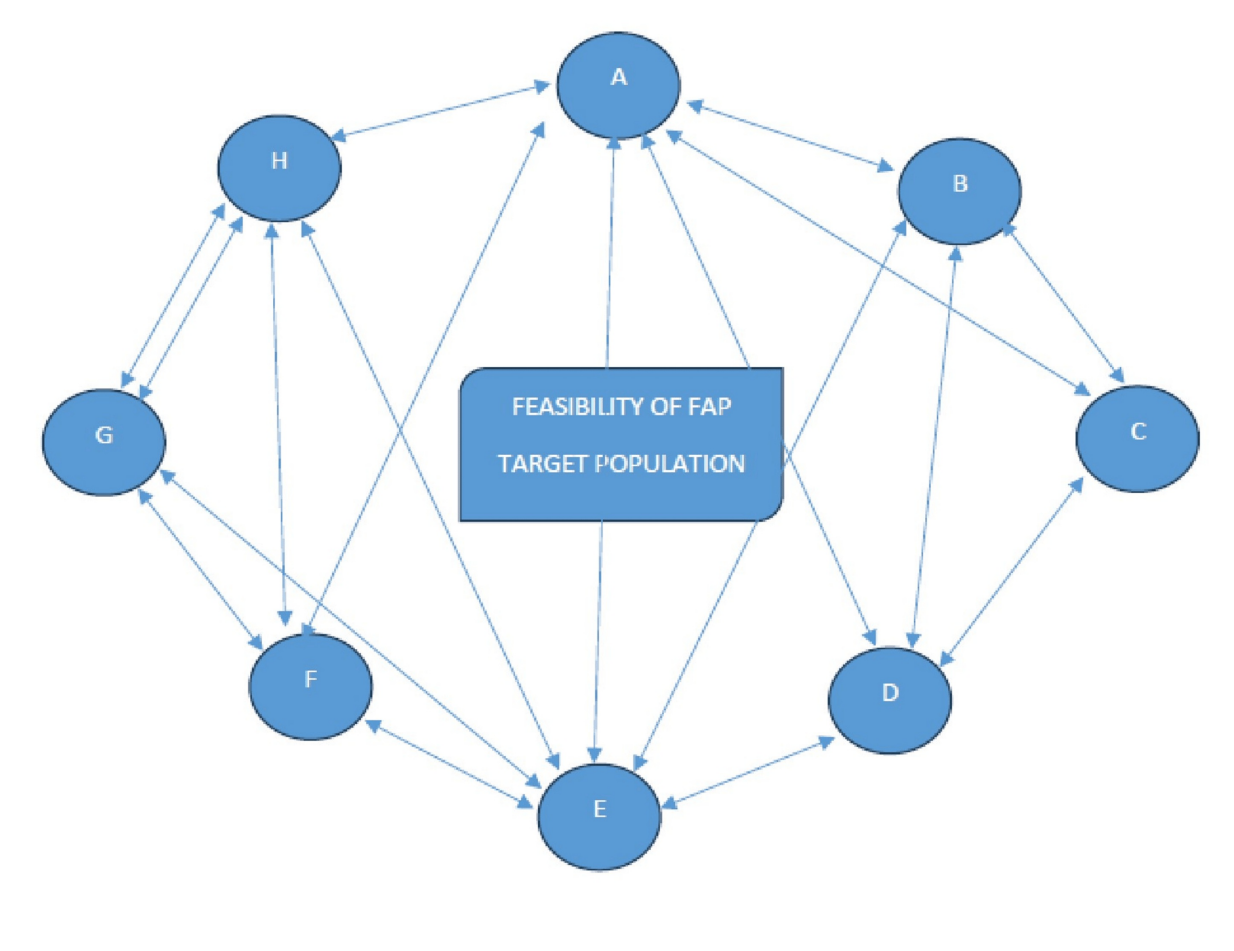
Sociogram of FGD analysis of the families adopted under the FAP. Letters A to H are the participants The alphabets A to H are the family members adopted by the students under the Family Adoption Program (FAP). FGD: Focus group discussion

Families prioritize on-site screening and basic treatment support, welcome the FAP visits, and want continuity and timely communication beyond scheduled encounters. The thematic analysis revealed that communities place strong value on access to basic diagnostics, expressing a clear need for point-of-care tools such as glucometer strips, hemoglobin testing, and blood pressure apparatus to support early detection of diabetes, anemia, and hypertension. They also preferred the inclusion of essential medicines, such as iron and folic acid (IFA) and calcium tablets, along with simple remedies for common minor illnesses, thereby linking the usefulness of the FAP to tangible health benefits. Participants described the program as highly acceptable and beneficial, particularly in areas with limited access to healthcare facilities, and they welcomed recurring, need-based visits. They also expected timely guidance beyond scheduled visits and structured follow-ups when abnormal findings were identified, indicating a preference for a relationship-based model of continuous care. These insights highlight the need to integrate basic screening services, provide limited essential medicines under proper supervision, formalize communication and follow-up processes, and strengthen continuity of care to enhance trust and program impact. All of the above-mentioned findings are mentioned in Figure 6.

The in-depth interviews with families echoed many of the concerns raised during the focus group discussions with healthcare workers. A recurring issue was the lack of basic medical supplies during student visits. Several families expressed that they had expected simple diagnostic support such as glucometer strips, hemoglobin testing, or basic first-aid medicines, but these were often unavailable. Another common concern is related to follow-up. Many families reported that although students initially provided advice or referrals, they rarely returned to check on progress. This gap in continuity left families feeling uncertain and unsupported, mirroring similar observations from the Focus Group Discussion (FGD). Families also described challenges in navigating healthcare facilities. While they appreciated the guidance given by students, they often struggled to reach hospitals or complete referral processes on their own and felt that more hands-on assistance would have been helpful. Finally, several participants noted that visits were irregular or stopped after just one or two interactions. This inconsistency weakened trust and led families to feel abandoned, an issue that community members had also emphasized in the FGD. Overall, the in-depth interviews (IDIs) reinforced the need for consistent engagement, structured follow-up, and better logistical support within the FAP. The analysis of the study was done in terms of SWOC revealing major findings as mentioned in Table 1.

**Table 1:** SWOC analysis SWOC: Strengths, Weaknesses, Opportunities and Challenges

Strengths	Weaknesses
Strong faculty engagement	Workload concerns
Opportunities	Challenges
Community-health system linkages	Academic workload balance

## Discussion

This mixed-methods study comprehensively evaluated the FAP at a medical college in Assam, combining quantitative and qualitative data from students, faculty, healthcare workers, and families. Using a SWOC approach as mentioned before and stakeholder feedback, the study highlights FAP’s impact and operational realities. Among students and faculty, a high number of students reported FAP as their first major field exposure in alignment with other studies [5-8], with most interacting easily with adopted families. Faculty unanimously supported FAP, recognizing its alignment with CBME, though they noted challenges like increased workload and the need for better student preparation. Community health workers (ASHAs and ANMs) acknowledged the program’s role in raising preventive health awareness, emphasizing the importance of their active involvement for sustainability and effective referrals. Beneficiary families expressed high satisfaction in alignment with other studies [5-9] with FAP’s health education and student engagement. However, issues like irregular visits, inconsistent follow-up, and limited support in formal healthcare access persisted. Despite desires for comprehensive support, few families experienced behavioral changes or actionable referrals.

Limitations

There are a number of limitations to this study that could impact how its findings are interpreted and applied generally. First, the representativeness of various viewpoints may have been limited by the small sample sizes for focus groups and IDIs, especially among families, faculty, and medical professionals, gathered from a single institution. Given the favorable relationship observed between students and families, the use of self-reported data raises the possibility of recall and social desirability bias. Furthermore, the study focused on qualitative impressions and perceived benefits rather than objectively measuring changes in clinical or health service utilization. Feedback from instructors and students may also be biased, with respondents who are more motivated or involved being over-represented.

Triangulation

Triangulation in this study refers to validating the findings by comparing and integrating perspectives from families, healthcare workers, students, and faculty to obtain a comprehensive understanding of the FAP.

Families

Appreciated health checks, access to basic investigations, and trust-building efforts resulted from regular student visits. Families expressed high satisfaction and are open to ongoing participation.

Healthcare workers

Increased community awareness was observed but unmet material needs and difficulties in contacting students outside regular hours were highlighted.

Faculties

Faculties strongly endorsed community engagement but expressed a need for enhanced student training and monitoring to maximize program impact.

Students

Students demonstrated earnest participation, with the greatest engagement in the first and second years, and a preference for a manageable number of families per student.

Recommendations

Strengthening Training and Preparation

Structured training for students and faculty on community engagement, communication, cultural sensitivity, and preventive care to enhance effectiveness in the field should be provided.

Enhancing Logistical Support

Students should be equipped with essential medical kits (glucometer, BP monitor, medicines) and reliable transport should be ensured to facilitate smooth and meaningful community visits.

Improving Communication and Follow-up Systems

Clear protocols and digital tools for students, faculty, health workers, and families to maintain continuous contact, timely follow-up, and emergency response should be established. 

Fostering Community and Administrative Involvement

Local leaders and community health workers should be engaged actively to increase program ownership, coordination, and sustainability within the community.

Institutionalizing Feedback and Monitoring

Regular feedback mechanisms from all stakeholders (students, families, faculty, health workers) should be implemented to evaluate progress and adapt the program iteratively for better outcomes.

Table 2 summarizes the entire findings of FGDs and IDIs.

**Table 2:** Summary & recommendations based on both IDI + FGD findings IDI: In-Depth Discussion; FGD: Focus Group Discussion

What Worked?	What Needs Improvement?	Recommendations
Positive attitude and respectful behavior of students	Lack of follow-up	Supply basic diagnostic tools (Hb strips, glucometers)
Useful health education (especially on hygiene, MCH)	No guidance in accessing the hospital or formal care	Include a navigation aid/referral system
Families want continued association with students.	Perceived superficial engagement if visits are infrequent	Assign fixed families for long-term engagement

## Conclusions

The FAP is a wonderful initiative that brings medical education closer to the community. It has seen enthusiastic participation from both faculty and students, and the positive responses from local families show that it’s making a real difference. By connecting students directly with families, the program helps them understand the many social factors that affect health something textbooks alone can’t teach. Some students find language or cultural differences a barrier when communicating with families, and better training could ease this. These continuous connections are crucial for lasting impact but need more structure to thrive. With some focused improvements like better preparation before fieldwork, streamlined logistics, and support to keep the bonds strong, FAP can grow even stronger. With commitment from the college, this program has the potential to become a shining example across India of how medical education can truly bridge the gap between classrooms and communities, preparing doctors who not only treat illnesses but also understand the people behind them.

## Appendices

Questionnaire for MBBS students

Section A: Background

1. Year of study: ( ) 1st ( ) 2nd ( ) 3rd 2. Number of FAP visits completed: ( ) <5 ( ) 5-10 ( ) >10 3. Number of families adopted: ( ) 1 ( ) 2-3 ( ) 4-5 ( )>5 4. Was this your first exposure to field-based health work? ( ) Yes ( ) No

Section B: Experience and Learning 5. Was your communication with the family members smooth? ( ) Yes ( ) No ( ) Sometimes 6. Did you face any language or cultural barriers? ( ) Yes ( ) No 7. Rate your learning (1-Poor to 5-Excellent): a. Communication skills 1 = Poor, 2 = Fair, 3 = Good, 4 = Very Good, 5 = Excellent b. Understanding social determinants of health 1 = Poor, 2 = Fair, 3 = Good, 4 = Very Good, 5 = Excellent c. Empathy and professionalism 1 = Poor, 2 = Fair, 3 = Good, 4 = Very Good, 5 = Excellent d. Teamwork 1 = Poor, 2 = Fair, 3 = Good, 4 = Very Good, 5 = Excellent

Section C: Programme Feedback 8. Ideal number of families to adopt per student: ( ) 1 ( ) 2-3 ( ) 4-5 9. Frequency of visits felt appropriate? ( ) Yes ( ) No ( ) Could be improved 10. Should FAP be mandatory for all students? ( ) Yes ( ) No Optional 11, Does FAP hamper your routine classes? ( ) Yes ( ) No 12. Do you like being part of FAP? ( ) Yes ( ) No If no? why?.................................................................................................................................. 13. Do you think FAP will help you connect more with the society as a health care professional? ( ) Yes ( ) No
